# Observed hydrological changes associated with active tectonic blocks before three consecutive earthquakes in Qinghai, China

**DOI:** 10.1038/s41598-023-36274-2

**Published:** 2023-06-02

**Authors:** Huaizhong Yu, Lei Liu, Yuchuan Ma, Rui Yan, Jie Liu, Yawei Ma, Zeping Li, Xiaotao Zhang, Jing Zhao, Chen Yu

**Affiliations:** 1 China Earthquake Networks Center, Beijing, 100045 China; 2 Qinghai Earthquake Agency, Xining, 810001 China

**Keywords:** Hydrology, Natural hazards, Solid Earth sciences

## Abstract

In the past 2 years, three earthquakes of magnitude 6.0 and above occurred consecutively in Qinghai province, China, i.e., the 22 May 2021 *M*_s_7.4 Maduo, 8 January 2022 *M*_s_6.9 Menyuan, and 26 March 2022 *M*_s_6.0 Delingha earthquakes. The hydrological observation instruments set up by the China Earthquake Administration allow us to study the dynamic processes in the well-aquifer systems during the establishment of criticality. Particularly, the observations played an important role in the prediction of the 8 January 2022 *M*_s_6.9 Menyuan earthquake that was approved by the People’s Government of Qinghai province. This work presents the hydrological data recorded by 7 stations to show the short-term anomalies before these earthquakes. To explore the performance of the hydrological observations in detecting earthquakes that occurred on different active tectonic blocks, we calculate the relative amplitudes of the pre-seismic changes. Results indicate that markedly pre-seismic change is found if the observation station and the detection earthquake are on the same block, and moderate change is found if they are on the adjacent blocks, while the precursor is hard to be identified if they are on the separated blocks. The variations in the hydrological responses may be caused by the strength weakening (or dilatancy) of source media. And the increased volumes in the crust can be evidenced by the changes in the geodetic time series in the same neighborhoods and during the same period, augmenting stress loading between the blocks.

## Introduction

Earthquake prediction in China is mainly based on empirical methods, in which nationwide observation stations are set up to monitor earthquake-related information. On 8 January 2022, the *M*_s_6.9 Menyuan earthquake (MYEQ) occurred in Qinghai province, China. The earthquake has been predicted by China Earthquake Administration (CEA) in both short and medium-to-long terms^1^. The Qinghai Earthquake Agency, CEA submitted reports to the People’s Government of Qinghai province on December 30 and 31, 2021. They predicted that an earthquake of magnitude 6–7 would occur in Qinghai soon, and suggested measures to deal with the earthquake. This is a meaningful practice, in which the hydrological observations in Qinghai province played an important role.

Earthquake-related hydrological observations can reveal the change of regionally tectonic activities and indicate the potential of a future earthquake^2^. Many studies of earthquake precursor monitoring are carried out based on this thought. For example, Roeloffs^3^ summarized the hydrologically pre-seismic changes of earthquakes in China. Kissin et al.^4^ and King et al.^5^ reported respectively the short-term precursors in Turkmenistan and Japan. Skelton et al.^6^ showed the changes in hydrochemistry before the earthquakes in Iceland. Moreover, Cicerone et al.^7^ suggested that the amplitude of a hydrologically pre-seismic anomaly is decided by the magnitude of the detection earthquake and distance from the epicenter^8,9^. The studies have indicated that the accumulation of tectonic stress is one of the major mechanisms to create hydrological changes^10–12^. A large earthquake is usually regarded as the rupture of the lithosphere in the upper crust subjected to long-term tectonic stress^13,14^. If the source media are loaded to the critical state, an earthquake may occur^15^. During the establishment of criticality, the permeability of source media increases with the development of cracks^16–18^. Since the depth of groundwater can reach 15–20 km^19,20^, the variation of permeability in the crust may lead to anomalous fluctuation in the hydrological system^21,22^.

However, we found that the hydrological changes before the MYEQ are hard to be explained just by using the magnitude-space relationship between the observation station and an earthquake of magnitude 6–7. Marked differences exist in the amplitudes of the changes between the stations which are not far from each other. Moreover, besides the MYEQ, the 22 May 2021 *M*_s_7.4 Maduo earthquake (MDEQ) and the 26 March 2022 *M*_s_6.0 Delingha earthquake (DLHEQ) also occurred in Qinghai province. The comparison of the performances of the hydrological observations before these earthquakes makes the problem stand out more clearly. Tectonically, the Qinghai region consists of complex systems of active faults. The hydrologically pre-seismic changes which are determined by the stress state of source media should be closely related to regional active tectonics^23,24^.

The physical and mechanical heterogeneities in each of the active tectonics in Qinghai province have been delineated by the active tectonic blocks^25^ which were derived from the spatial distribution of strong earthquakes and GPS velocity fields^26,27^. Based on the active tectonic block theory^28^, the formation mechanism of strong earthquakes has been established^29,30^. The stress loading between the blocks is not only from the movement of blocks but also from their deformation^31^.

This work attempts to study the prediction performances of the hydrological changes before the MDEQ, MYEQ, and DLHEQ and the modulation imposed by the active tectonic blocks. To show the validity of the results, the geodetic time series observed in the same neighborhoods and during the same period are also displayed. This paper includes seven sections. In addition to the introduction section, the “Regional tectonic setting” section shows the regional active tectonics of the study area; the “Hydrological observations” section is about the observation stations and the changes before the MDEQ, MYEQ, and DLHEQ, the “Methods” section introduces the methodology for relative amplitude calculation of the pre-seismic changes, the “Model of hydrological response” section suggests the models of the hydrological responses to earthquakes occurring on different blocks, and in the “Discussion” and “Conclusions” we will present some discussions and conclusions.

### Regional tectonic setting

**Figure 1:**
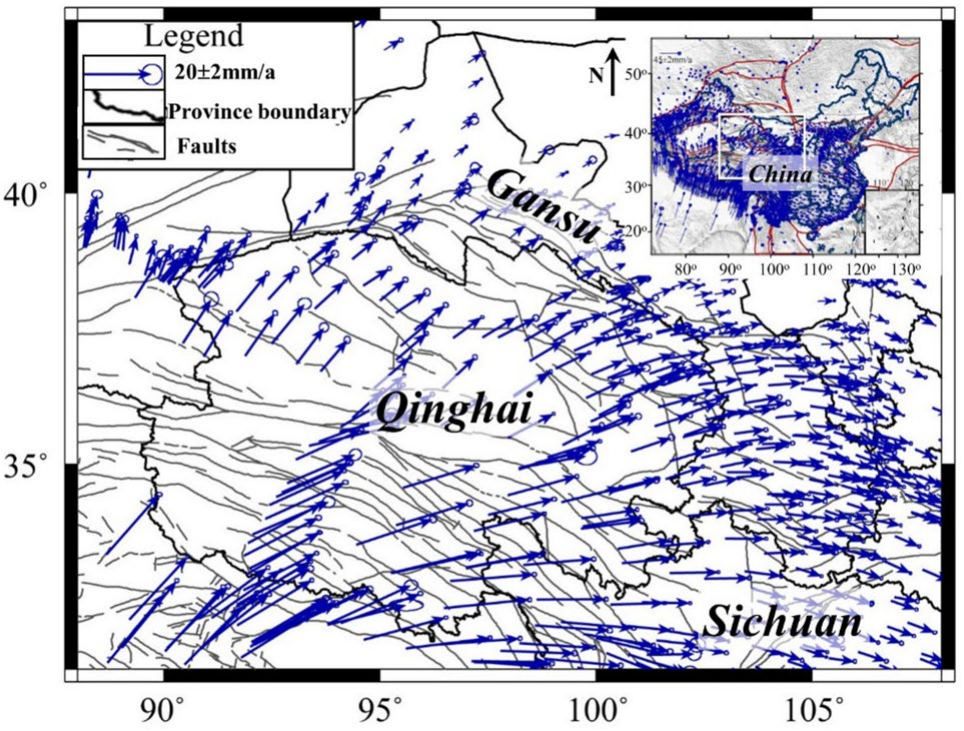
GPS velocity field in Qinghai province and its adjacent areas during 1991–2016. The data was retrieved from Wang and Shen^27^. The figure was generated by using the Generic Mapping Tools (GMT) 4 (https://www.generic-mapping-tools.org/download/, accessed on 27 January 2022).

**Figure 2:**
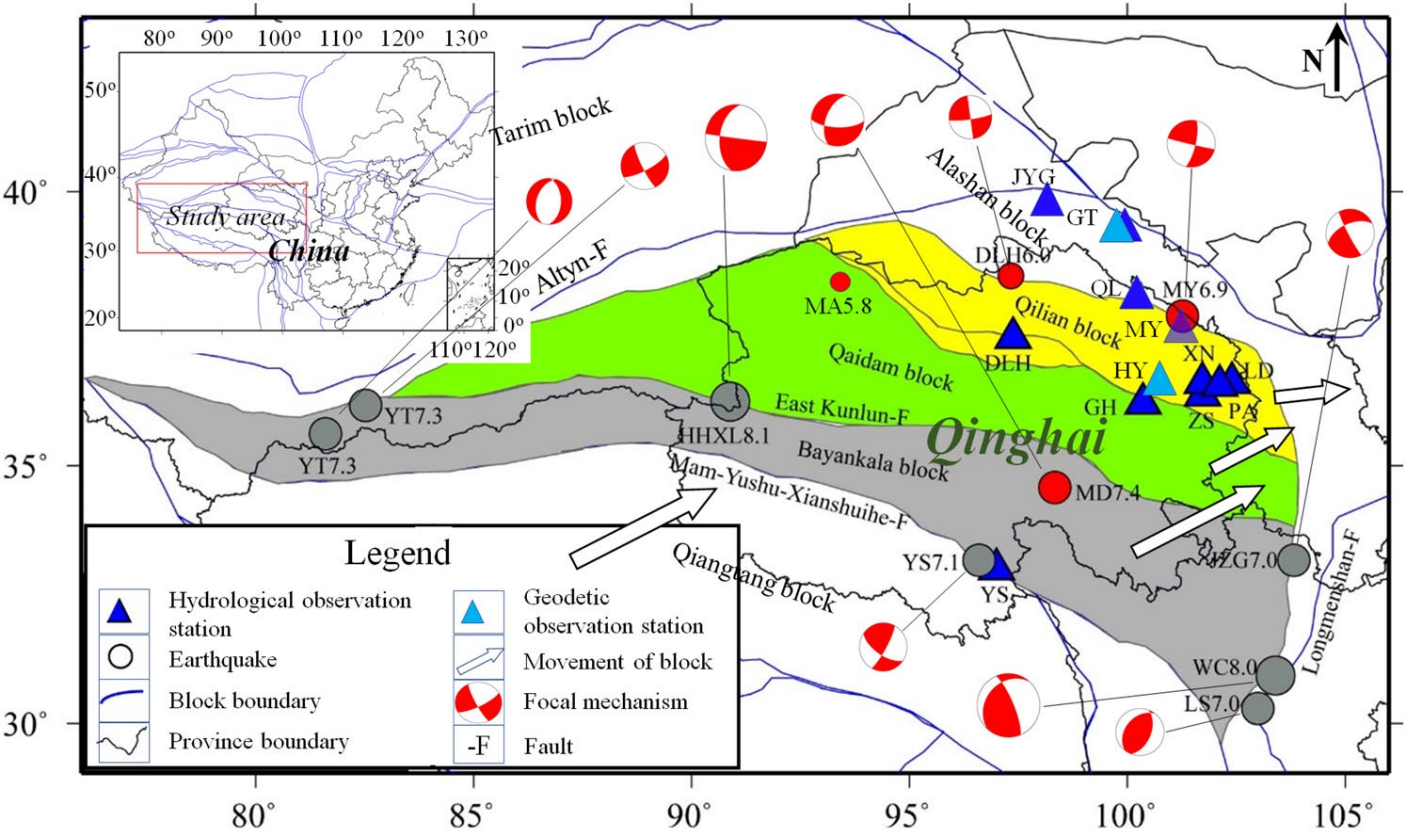
The active tectonic blocks, observation stations, and studied earthquakes in Qinghai province. The Bayan Har, Qaidam, and Qilian blocks are marked with grey, green and yellow, whose movements are indicated by hollow arrows. The earthquakes of magnitude 7.0 and above occurred on the Bayan Har block and the *M*_s_ ≥ 6.0 earthquakes in Qinghai province since 2021 are displayed with gray and red circles. Also shown in the Figure are the 7 hydrological and 2 geodetic observation stations discussed in this work. *Earthquakes*: HHXL8.1: 14 November 2001 *M*_s_8.1 HohXil, YT7.3: 21 March 2008 *M*_s_7.3 Yutian, WC8.0: 12 May 2008 *M*_s_8.0 Wenchuan, YS7.1: 14 April 2010 *M*_s_7.1 Yushu, LS7.0: 20 April 2013 *M*_s_7.0 Lushan, YT7.3: 12 February 2014 *M*_s_7.3 Yutian, JZG7.0: 8 August 2017 *M*_s_7.0 Jiuzhaigou, MD7.4: 22 May 2021 *M*_s_7.4 Maduo, MY6.9: 8 January 2022 *M*_s_6.9, and DLH6.0: 26 March 2022 *M*_s_6.0 Delingha. *Hydrological observation stations*: DLH: Delingha, GH: Gonghe, GT: Gaotai, JYG: Jiayuguan, LD: Ledu, MY: Menyuan, PA: Pingan, QL: Qilian, XN: Xining, YS: Yushu, and ZS: Zuoshu. *Geodetic observation station*: GT: Gaotai, and HY: Huangyuan. The figure was generated by using the Generic Mapping Tools (GMT) 4 (https://www.generic-mapping-tools.org/download/, accessed on 27 January 2022).

Qinghai province is located on the northeastern margin of the Qinghai-Tibet Plateau. GPS velocity measurements show that, due to the intensive extrusion of the Indian plate and the interaction between the Qinghai-Tibet plateau, Alashan block, and Ordos block, Qinghai province mainly moves along the *NEE* direction^27^. The velocity decreases significantly from southwest to northeast, with a maximum of 25.0 mm/a at the southwest and a minimum of 10.02 mm/a at the east (Fig. 1). In terms of the study of Zhang et al.^25^, Qinghai province can mainly be divided into the Bayan Har, Qaidam, Qilian, and Qiangtang blocks (Fig. 2). Due to the lack of hydrological observation station on the Qiangtang block, the block is not considered in this study.

The southernmost block is the Bayan Har block, which moves along the direction of *NE*61^o^ with a velocity of 21 mm/a. The southern and northern boundaries of this block are the Mani-Yushu-Xianshuihe and eastern Kunlun faults, respectively, which are all dominated by the sinistral strike-slip. The eastern boundary is the Longmenshan fault zone with a thrust velocity of 0.6–1.2 mm/a^32,33^, and the western boundary is composed of the southwest segment of the Altyn fault. On this block, the 14 November 2001 *M*_s_8.1 HohXil, 21 March 2008 *M*_s_7.3 Yutian, 12 May 2008 *M*_s_8.0 Wenchuan, 14 April 2010 *M*_s_7.1 Yushu, 20 April 2013 *M*_s_7.0 Lushan, 12 February 2014 *M*_s_7.3 Yutian, 8 August 2017 *M*_s_7.0 Jiuzhaigou, and 22 May 2021 *M*_s_7.4 Maduo earthquakes occurred.

The north side of the Bayan Har block is the Qaidam block. The seismic activity on this block is markedly lower than that of the Bayan Har block, with an average of 0.15 earthquakes of magnitude 6.0 or above every year. The movement direction of the Qaidam block is the same as the Bayan Har block, with an average velocity of 12–14 mm/a.

The northernmost block is the Qilian block. This block moves almost in the direction of *EW*, with a speed of 7–14 mm/a. The MYEQ and DLHEQ occurred on this block.

### Hydrological observations

**Figure 3:**
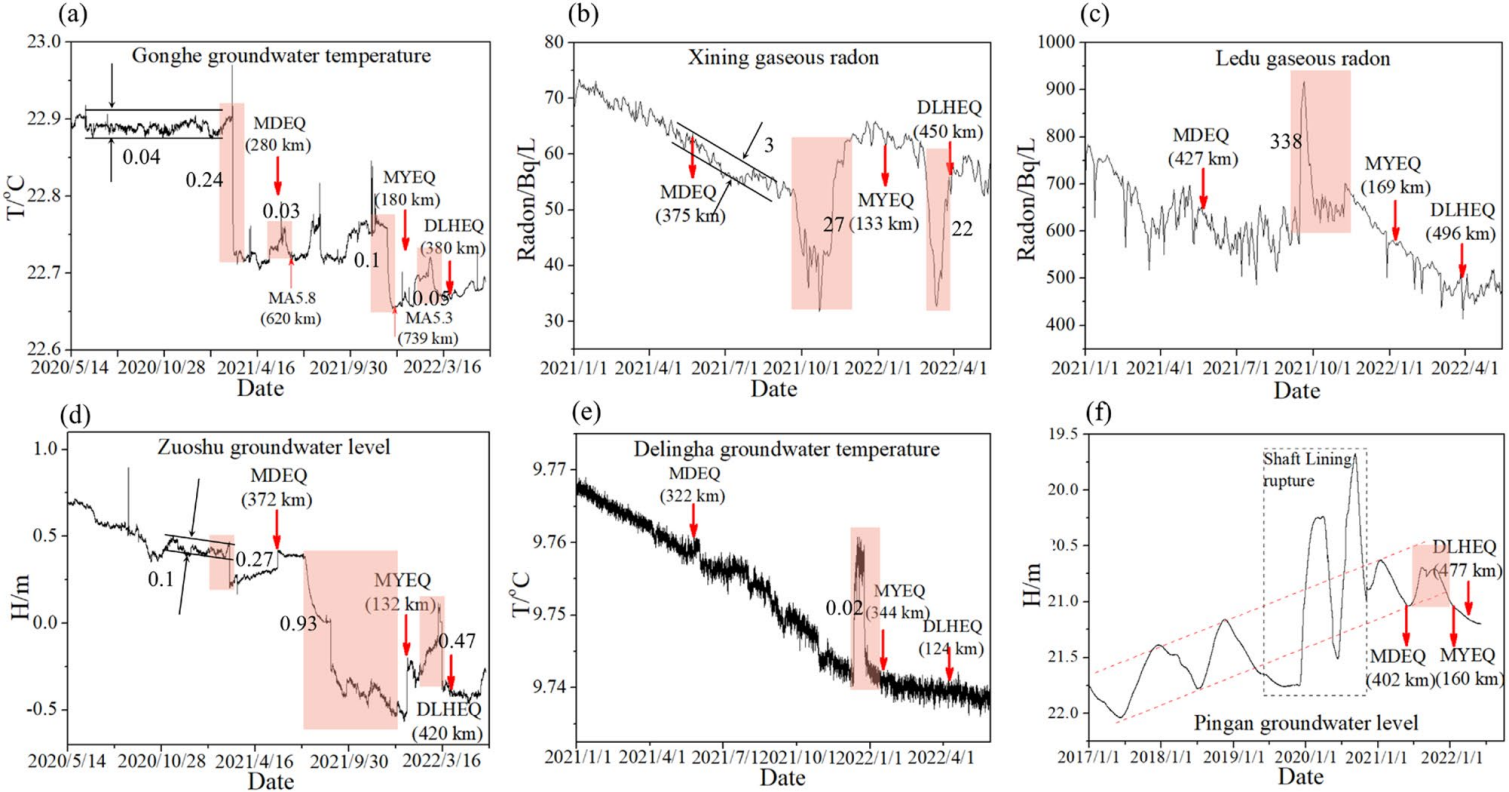
Hydrological data recorded by the stations in Qinghai province. The times of MDEQ, MYEQ, and DLHEQ in each of the maps are indicated by vertical arrows, while the corresponding pre-seismic changes are marked with red boxes. The numbers in brackets show the epicenter distances. (**a**) Gonghe groundwater temperature, MA5.8 and MA5.3: the 16 June 2021 Mangai, Qinghai *M*_s_5.8 and 19 December 2021 Mangai, Qinghai *M*_s_5.3 earthquakes. (**b**) Xining gaseous radon. (**c**) Ledu gaseous radon. (**d**) Zuoshu groundwater level. (**e**) Delingha groundwater temperature. (**f**) Pingan groundwater level. The amplitudes of background noises of the Gonghe groundwater temperature, Zuoshu groundwater level, and Xining gaseous radon are displayed in (**a**),(**b**), and (**d**).

**Figure 4:**
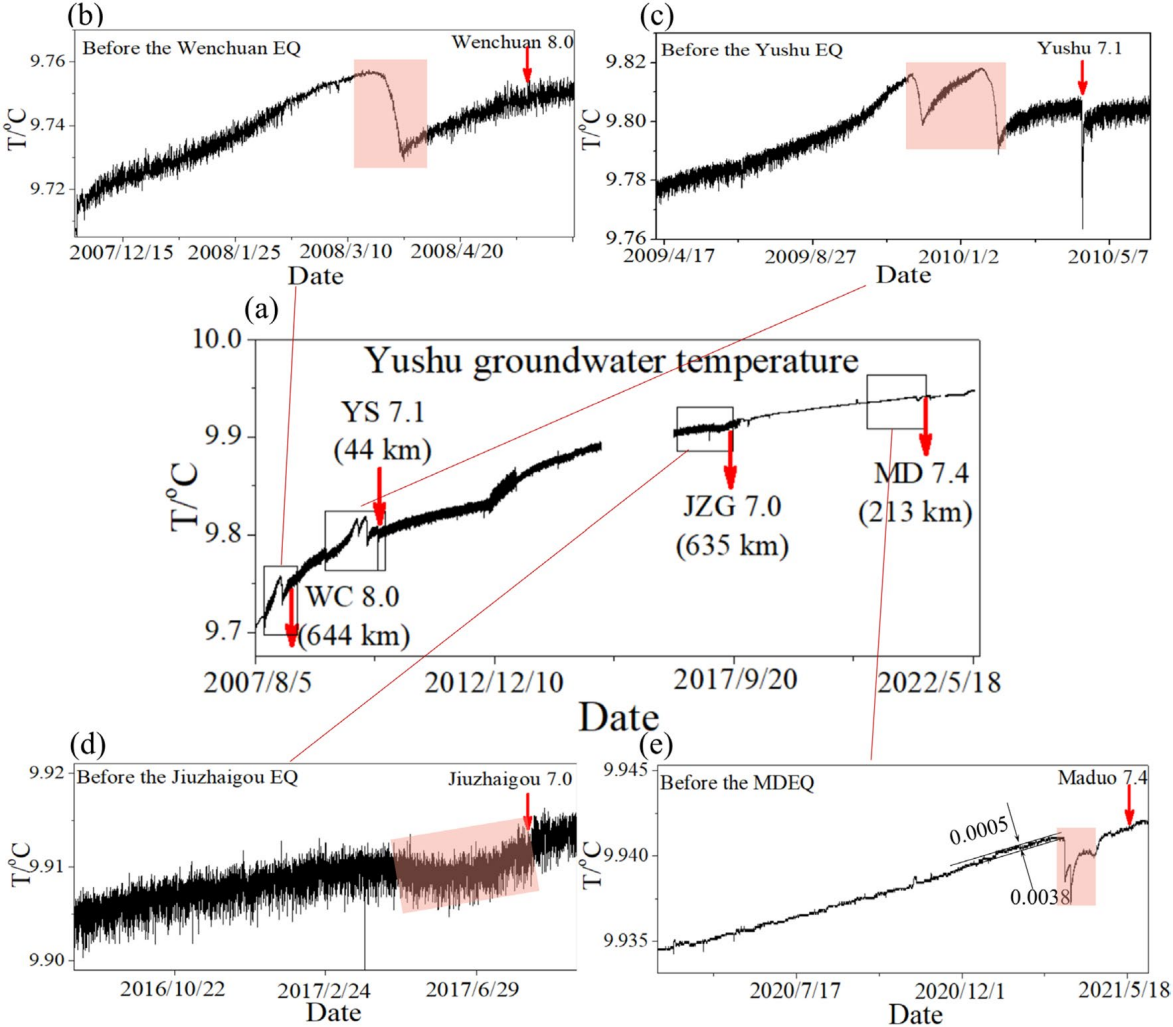
Time series of Yushu groundwater temperature (**a**) and its evolutions before large earthquakes. The changes before the 12 May 2008 *M*_s_8.0 Wenchuan, 14 April 2010 *M*_s_7.1 Yushu, 8 August 2017 *M*_s_7.0 Jiuzhaigou, and 22 May 2021 *M*_s_7.4 Maduo earthquakes are displayed in (**b**),(**c**),(**d**), and (**e**). The amplitudes of the anomaly and background noise before the MDEQ are also shown in (**e**).

In the past 2 years, the MDEQ, MYEQ, and DLHEQ occurred consecutively in Qinghai province. Fortunately, there are equipped with high-quality instruments, allowing us to study the dynamic responses in the hydrological system before and after the earthquakes. Figure 2 shows the location of the observation stations. They are distributed on the Qilian, Qaidam, and Bayan Har blocks, respectively. Detailed information about the stations is listed in Table 1. Figures 3 and 4 show the sequences recorded by each station, including the groundwater temperature at Gonghe, Delingha, and Yushu, the groundwater level at Zuoshu and Pingan, and the gaseous radon at Xining and Ledu.

**Table 1:** Detailed information on the hydrological observation stations.

No	Station	Observation	Active block	Bedrock	Depth (m)	Instrument	Resolution	Sampling frequency
1	YS	Temperature	Bayan Har	Granite	105	SWY-II	0.0001 °C	1-*h*
2	GH	Temperature	Qaidam	Siltstone	174	SZW-II	0.0001 °C	1-*h*
3	ZS	Water level	Qilian	Sandstone	107	SWY-2	0.001 m	1-*h*
4	PA	Water level	Qilian	mudstone	105	SZW-1A	0.001 m	1-*h*
5	XN	Gaseous radon	Qilian	Sandstone	261	DDL-1	0.1 Bq/L	1-*day*
6	LD	Gaseous radon	Qilian	Glutenite	1	SD-3A	0.01 Bq/L	1-*day*
7	DLH	Temperature	Qilian	Granitic gneiss	98	SZW-1A	0.0001 °C	1-*h*

During the preparation for a large earthquake, the changed permeability derived from the variation of cracks in the crust may lead to anomalous fluctuation in the hydrological system. If the observation value of a station significantly exceeds the background noise, it could be regarded as an anomaly. We adopted the difference between the maximum and minimum values of the detrend data within a timeframe of at least 3 months before an anomaly as the background noise (Fig. 3) that should come from the instrument or tectonic activity. Due to the location difference between the observation station and the detected earthquake, the amplitude of anomalies before earthquakes may be changed by the tectonic block modulation, but the background noise is unaffected.

In Fig. 3a, the Gonghe groundwater temperature dropped rapidly by 0.24 °C in March 2021, about 60 days before the MDEQ, with an epicenter distance of 280 km. Then, in December of the same year, a smaller decrease of 0.1 °C was observed again, and the MYEQ occurred 25 days later, with an epicenter distance of 180 km. Thereafter, in February 2022, about 30 days before the DLHEQ, the temperature sharply dropped (~ 0.05 °C) for the third time, with an epicenter distance of 380 km.

Similar anomalies before the MDEQ, MYEQ, and DLHEQ can also be found in the time series of the Zuoshu groundwater level (Fig. 3d). It dropped by 0.27 m on 25 February 2021, 86 days before the MDEQ, with a distance of 372 km; 0.93 m on 25 August 2021, 136 days before the MYEQ and 132 km from the epicenter; and 0.47 m on 10 March 2022, 16 days before the DLHEQ, with the distance of 420 km.

There were two significant reductions in the time series of the Xining gaseous radon, both of which exceeded 20 Bq/L (Fig. 3b). One is 61 days before the MYEQ, and the other occurred 15 days before the DLHEQ. The distances to the epicenter of these two earthquakes are 133 and 450 km, respectively. In addition, the Ledu gaseous radon, Delingha groundwater temperature, and Pingan groundwater level showed uplift or anomalous annual variation about 94, 37, and 89 days before the MYEQ (Fig. 3d–f), with the epicenter distances of 169, 344, and 160 km. The Yushu groundwater temperature dropped by 0.0038 °C on 15 March 2021, about 32 days before the MDEQ (Fig. 4e), with an epicenter distance of 214 km. Although the fluctuation amplitude was significantly reduced due to the instrument maintenance after the Jiuzhaigou earthquake, the relative amplitude was relatively higher.

To display the pre-seismic changes, we provide observation sequences for about a year before the mainshocks in Fig. 3. The Zuoshu water level and Gonghe water temperature started on 20 May 2020 (i.e., a year before the MDEQ), Delingha water temperature, Xining and Ledu gaseous radon began on 1 January 2021 (i.e., a year before the MYEQ), and Pingan water level was from January 2017 to manifest anomalous annual changes before the MYEQ. Due to the different spatial locations and tectonic environments of observation stations, there are differences in the timing of corresponding anomalies, which could be several days to years before the mainshocks.

## Methods

Comparing the hydrological data observed by different stations (Figs. 3 and 4), it is clear that they all detected markedly pre-seismic anomalies. Some observations, such as the Gonghe groundwater temperature, correlate well with the magnitude-space relationship between the station and the mainshock (Fig. 3a). The distances to the *M*_s_7.4 MDEQ, *M*_s_6.9 MYEQ, and *M*_s_6.0 DLHEQ are respectively 180, 280, and 380 km, and the corresponding amplitudes of pre-seismic changes are 0.24, 0.1, and 0.05 °C. More detailed comparison of the pre-seismic changes shown in Fig. 3, however, we notice a significant difference. Although the Zuoshu station is located just about 120 km east of the Gonghe (Fig. 2), the amplitudes of the observed changes before the MDEQ, MYEQ, and DLHEQ (Fig. 3d), which are respectively 0.27, 0.93, and 0.47 m, seem to be different from that of the Gonghe groundwater temperature. The amplitude before the MDEQ is lower than that before the other two earthquakes, even though the distance to the MDEQ (~ 372 km) is shorter than that to the DLHEQ (~ 420 km).

To investigate the performance of the hydrological observations, we calculate the relative amplitude for each pre-seismic change by using the following equation:

1 $$ R_{A} = \frac{{V_{A} }}{{V_{T} }}, $$

where *V*_A_ and *V*_T_ represent respectively the amplitudes of the pre-seismic change and its background noise.

For example, before the MDEQ, the amplitudes of the precursory change and background noise of Gonghe groundwater temperature are 0.24 and 0.04 °C (Fig. 3a). Thus, we can get its relative amplitude (*R*_A_) is 6.0.

**Figure 5:**
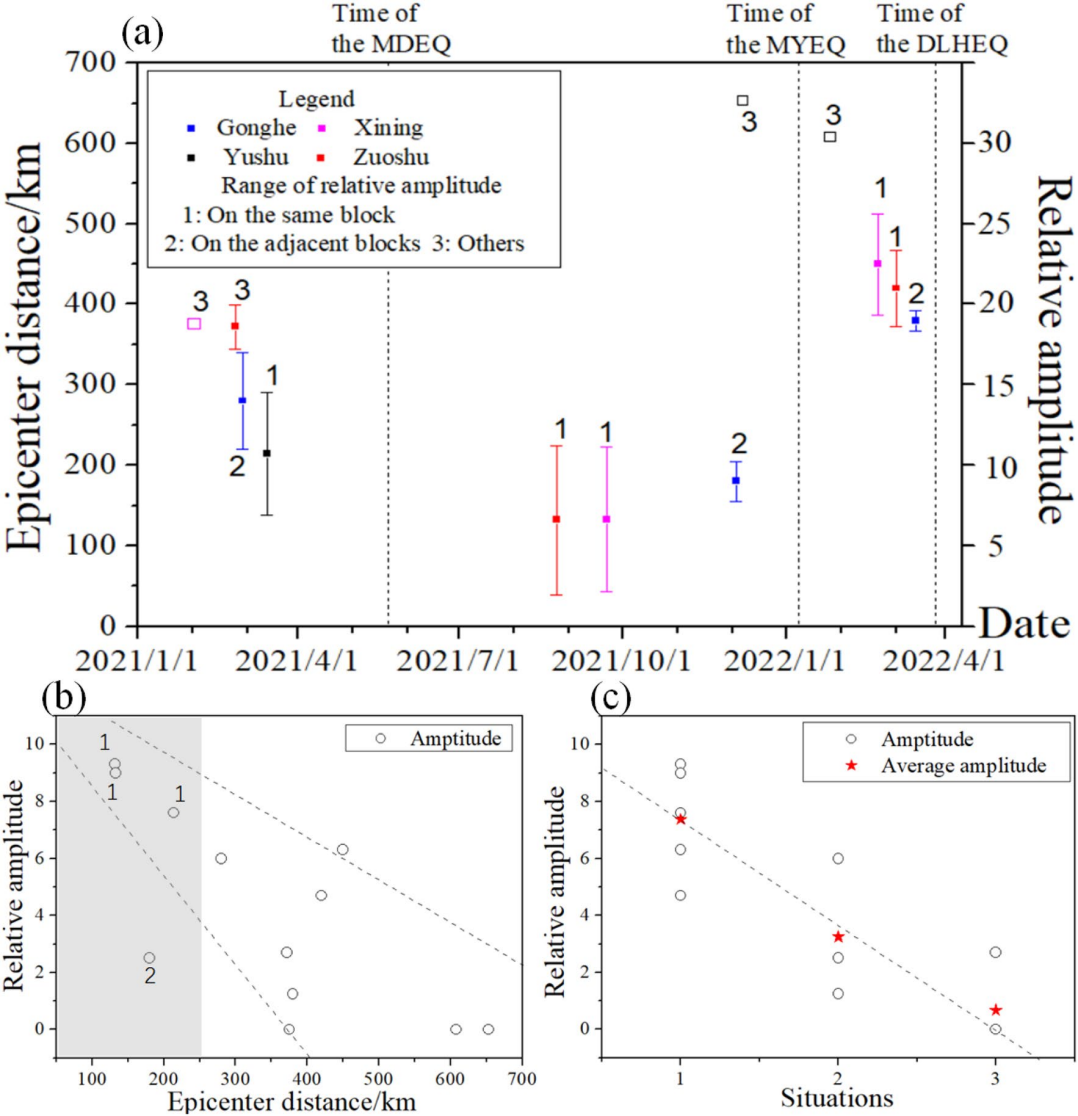
The influences of active tectonic blocks and epicenter distances on the relative amplitudes of pre-seismic changes. (**a**) Illustration of the relative amplitudes of the anomalous changes before the MDEQ, MYEQ, and DLHEQ observed by the Zuoshu groundwater level, Gonghe groundwater temperature, Xining gaseous radon, and Yushu groundwater temperature (expressed with red, blue, purple, and black). The right axis labels the relative amplitude, while the left axis indicates the distance to the epicenter of each mainshock, with the time of the anomaly displayed by the horizontal axis. Meanwhile, the situations for the relationship between the detection earthquake and each observation station are listed with numbers 1–3. Situation **1**: The station and earthquake are on the same active tectonic block, situation **2**: on the adjacent blocks, and situation **3**: others. (**b**) Statistics of relative amplitude changing with epicenter distance. The grey shows the area with epicenter distances less than 250 km, in which the situation for each observation station is labeled. (**c**) Variation of relative amplitudes with the situations of 1–3. The red pentagrams represent the average amplitude in each situation.

Figure 5a shows the relative amplitudes derived from the pre-seismic changes displayed in Figs. 3 and 4. Since the Delingha groundwater temperature, Pingan groundwater level, and Ledu gaseous radon exhibited anomalies just only before the MYEQ, it is unnecessary to include their relative amplitudes. Before the MDEQ, MYEQ, and DLHEQ, the relative amplitudes produced by the Gonghe groundwater temperature are 6.0, 2.5, and 1.25, and those produced by the Zuoshu groundwater level are roughly 2.7, 9.3, and 4.7 (Fig. 5a). For the detection of MYEQ, the relative amplitude of the Zuoshu groundwater level was much larger than that of the Gonghe groundwater temperature, while before the MDEQ, the situation is quite different. In addition, for some observations such as the Xining gaseous radon, significant anomalies could be found before the MYEQ and DLHEQ, but could not be found before the MDEQ, even though the distance to the MDEQ (~ 375 km) is less than that to the DLHEQ (~ 450 km).

The epicenter distances of each observation station are also shown in Fig. 5a. With the increase of epicenter distance, the relative amplitude of the observation stations decreases (Fig. 5b). However, there are significant differences in the statistics, such as the stations in the gray area of Fig. 5b. Apart from that, as shown in Fig. 4, the Yushu groundwater temperature changed anomalously not only before the MDEQ but also, within a short time, before several other earthquakes that occurred on the Bayan Har block, such as the 12 May 2008 *M*_s_8.0 Wenchuan, 14 April 2010 *M*_s_7.1 Yushu, and 8 August 2017 *M*_s_7.0 Jiuzhaigou earthquakes. Nevertheless, the MYEQ, whose magnitude and epicenter distance are almost the same as the Jiuzhaigou earthquake, was not detected by this station.

In addition to the epicenter distance, the active tectonic blocks should have some impact on the performance of the hydrologically pre-seismic changes. Tectonically, the Yushu station is located on the Bayan Har block, while the Gonghe station is on the Qaidam block (Fig. 2). Although the location of Zuoshu is very close to the Gonghe station, this station, as well as the Pingan, Ledu, and Delingha stations, is on the Qilian block. On the other hand, the detection earthquakes, i.e., the MDEQ, MYEQ, and DLHEQ, are distributed on the Bayan Har and Qilian blocks. From the statistical results in Fig. 5c, it can be seen that the relative amplitude of the pre-seismic change is very significant when the observation station and the detection earthquake are on the same block (situation 1); smaller amplitude is observed when they are on the adjacent blocks (situation 2), and the precursor can hardly be identified under the situation 3.

## Model of hydrological response

**Figure 6:**
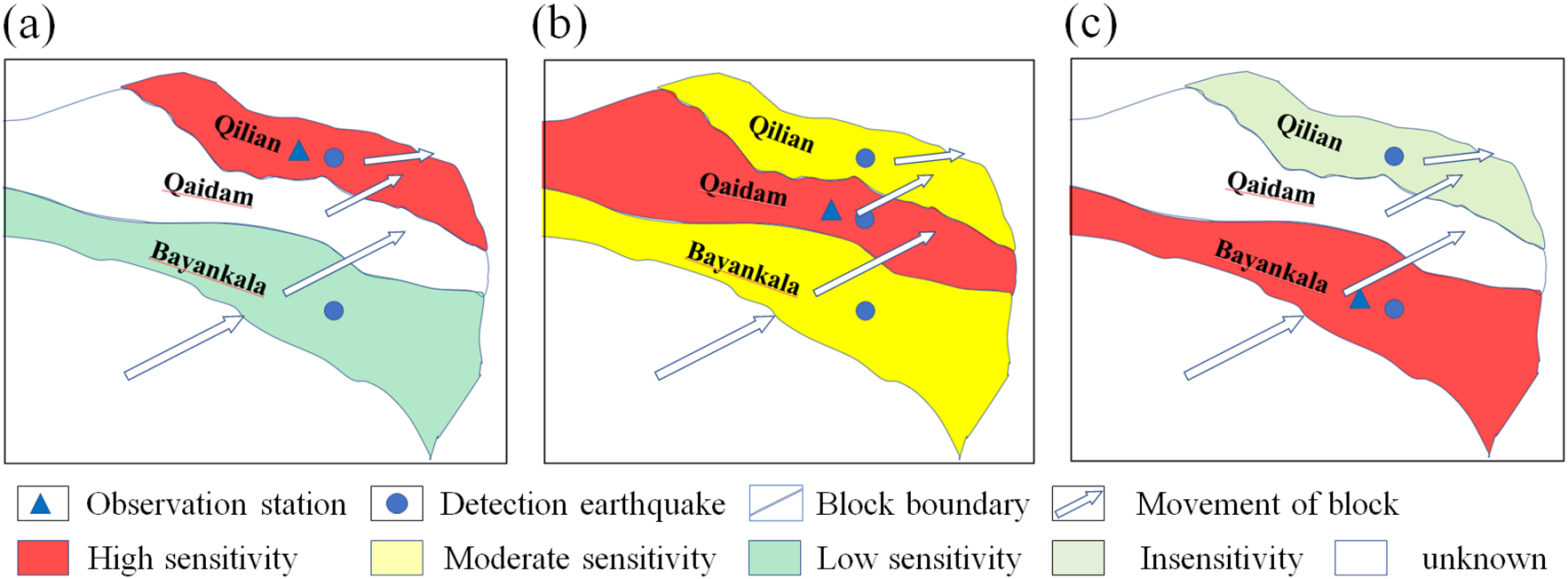
Model of hydrological response of an observation station to earthquakes occurring on different blocks. (**a**) The observation station is on the Qilian block. Red represents the earthquake that occurred on the Qilian block. The sensitivity of the response is high. Light blue denotes the earthquake that occurred on the Bayan Har block, and the sensitivity is low. (**b**) The observation station is on the Qaidam block. Red: the earthquake occurred on the Qaidam block. Yellow means the earthquake that occurred on the Qilian or Bayan Har blocks, and moderate sensitivity is observed. (**c**) The observation station is on the Bayan Har block. Red: the earthquake occurred on the Bayan Har block. Light gray: the earthquake occurred on the Qilian block, which is hardly detected by this station. White is for the earthquake that occurred on the Qaidam block, whose sensitivity is unknown.

We attempt to establish a model to connect the performance of the hydrologically pre-seismic changes with earthquakes occurring on different blocks (Fig. 6).

When an observation station is located on the Qilian block (Fig. 6a), it is very sensitive to earthquakes occurring on the same block. This can be approved by the significant anomalies before the MYEQ recorded by the Zuoshu groundwater level, Xining gaseous radon, Pingan groundwater level, Ledu gaseous radon, and Delingha groundwater temperature. Oppositely, the performance for predicting earthquakes that occurred on the Bayan Har block is low. We note that just the Zuoshu groundwater level manifested some short-term changes before the MDEQ (Fig. 3).

When the station is on the Qaidam block (Fig. 6b), it can detect earthquakes on its adjacent blocks (the Qilian and Bayan Har blocks) with moderate sensitivity (Fig. 5c). The Gonghe groundwater temperature showed a clear short-term anomaly not only before the earthquake that occurred on the Bayan Har block (the MDEQ) but also before the earthquake on the Qilian block (the MYEQ and DLHEQ). However, the relative amplitude of the Gonghe groundwater temperature, before the MYEQ (~ 2.5), is much smaller than that of the Zuoshu groundwater level (~ 9.3). Moreover, the relative amplitude of the Gonghe groundwater temperature, for the prediction of MDEQ (~ 6.0), was lower than that of the Yushu groundwater temperature (~ 7.6). We note that the 16 June 2021 Mangai, Qinghai *M*_s_5.8 and 19 December 2021 Mangai, Qinghai *M*_s_5.3 earthquakes occurred on the Qaidam block during the research period. Before these 2 earthquakes, marked pre-seismic anomalies were observed in the Gonghe water temperature. For example, it dropped by 0.03 °C before the Mangai *M*_s_5.8 earthquake (Fig. 3a). However, the changes were not observed at the stations on other tectonic blocks (Fig. 3). The result further indicates the modulation of tectonic blocks on the hydrological changes.

When the station is on the Bayan Har block (Fig. 6c), such as the Yushu groundwater temperature, it can well detect earthquakes that occurred on the Bayan Har block but has virtually no response to earthquakes occurring on the Qilian block.

The model looks to correlate well with the hydrological observations presented in this study, however, we do not know how robust it is, because just limited data are available for the analysis. Especially on the Bayan Har block, just only the Yushu station keeps running. Although it is very sensitive to strong earthquakes that occurred on the Bayan Har block, the sensitivity to earthquakes occurring on the Qilian block is needed to be verified by more observations. In addition, the distance to the epicenter should be included in determining the relative amplitude of the pre-seismic changes. For example, when the observation station and the detection earthquake are on the same active tectonic block (e.g., the Zuoshu groundwater level and Xining gaseous radon before the MYEQ, Yushu groundwater temperature before the MDEQ), the epicenter distances can be divided into three levels (~ 130, 210, and 420 km), and the relative amplitudes (which are roughly 9.0, 7.5, and 5.0) decrease with the increased epicenter distance. Moreover, the Yushu groundwater temperature (Fig. 4) did not show any anomalous change before the 21 March 2008 *M*_s_7.3 Yutian and 12 February 2014 *M*_s_7.3 Yutian earthquakes with epicenter distances of 1440 and 1380 km (Fig. 2).

## Discussion

The high-quality hydrological observations in Qinghai province allow us to study the short-term precursors before large earthquakes. Note that all the pre-seismic changes have been validated by the CEA immediately after the occurrence. By comparing the observations with precipitation and air pressure, the meteorological interferences were eliminated. And by investigating the surface water level, groundwater exploitation, agricultural irrigation, pollution, and other artificial activities were excluded.

**Figure 7:**
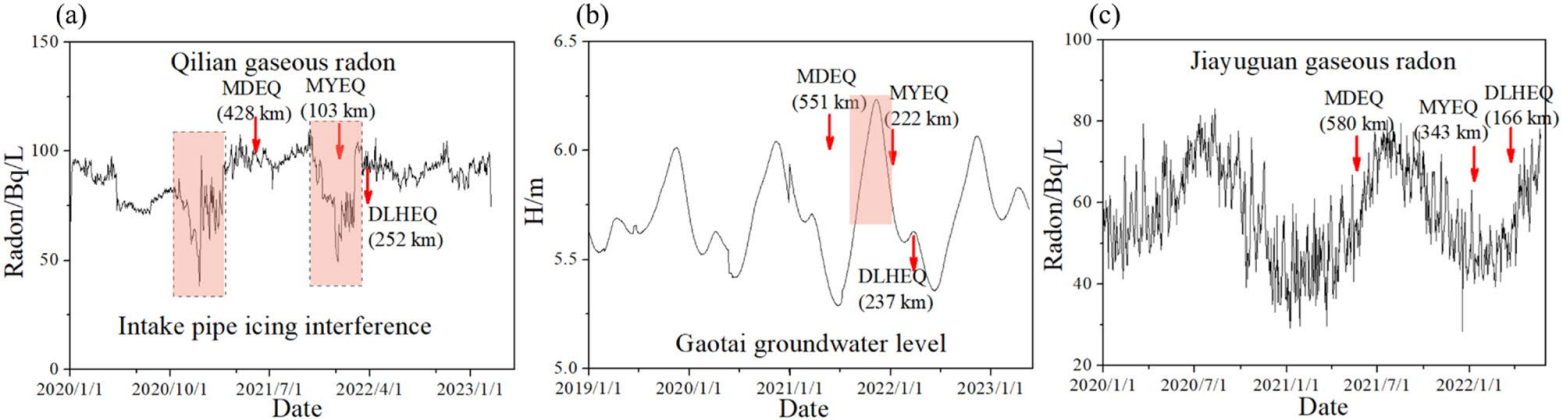
Hydrological data recorded by the stations on the north side of the MYEQ. (**a**) Qilian gaseous radon. (**b**) Gaotai groundwater level. (**c**) Jiayuguan Gaseous radon.

**Figure 8:**
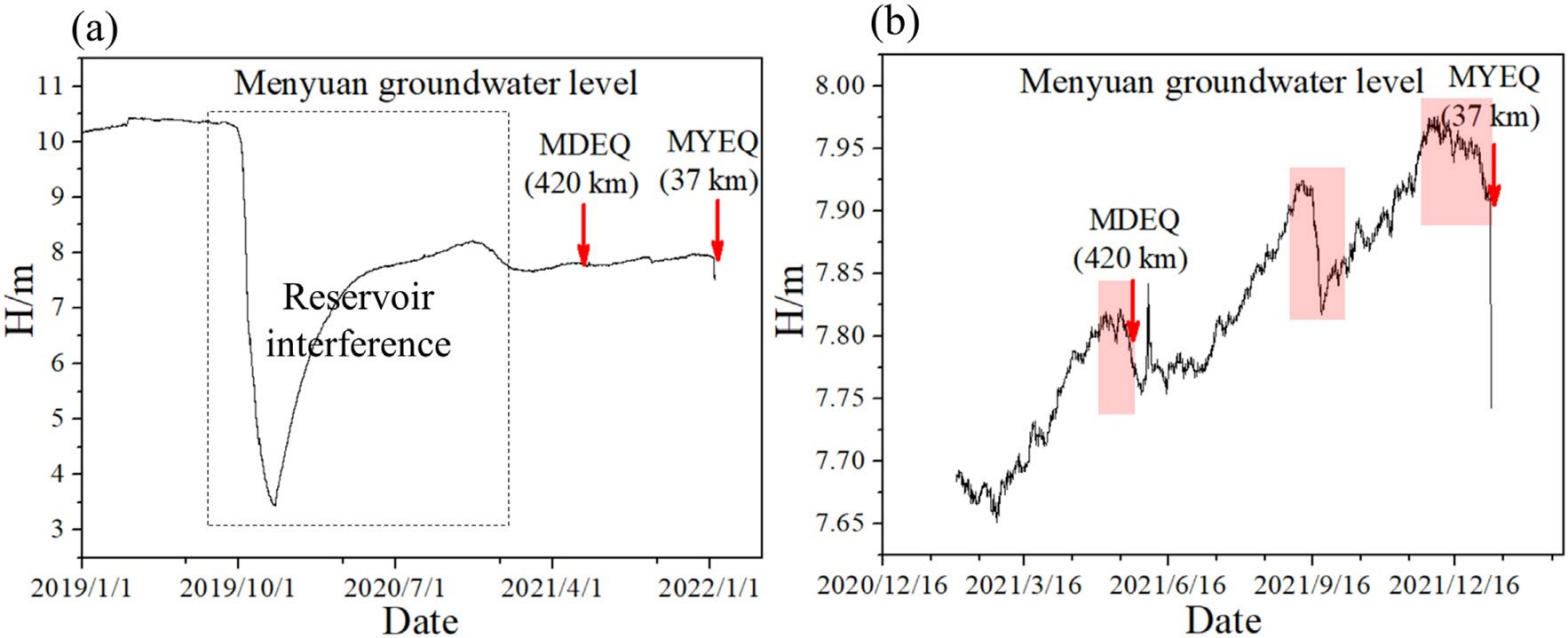
Time series of Menyuan groundwater level (**a**) and the short-term pre-seismic changes before the MDEQ and MYEQ (**b**).

In addition to the stations displayed in Fig. 3, there are four hydrological observations on the north side of the research area (i.e., the Qilian gaseous radon, Gaotai groundwater level, Jiayuguan gaseous radon, and Menyuan groundwater level)^34^. The corresponding sequences are shown in Figs. 7 and 8. Although these stations are not included in our study, the hydrologically anomalous changes correlate well with the logic rules declaimed by this manuscript. We note that the Menyuan and Qilian stations are located on the Qilian block, while the Gaotai and Jiayuguan stations are on the Alashan block (Fig. 2). It should be pointed out that the prediction performance of the Menyuan and Qilian observations is questionable due to the interferences induced by a reservoir nearby and the intake pipe icing. The Menyuan groundwater level detected short-term anomalies before the MDEQ and MYEQ (Fig. 8a). This station is in the border area between the Gansu and Qinghai provinces, about 37 and 420 km from the two epicenters. And the amplitude of the anomalies before the MYEQ is greater than the MDEQ (Fig. 8b). Similarly, significant drops were recorded before the MYEQ in the Qilian gaseous radon (Fig. 7a). On the other hand, for the stations on the Alashan block, smaller anomalies are observed (Fig. 8b) in the Gaotai groundwater level (with a 222 km epicenter distance) and are not observed (Fig. 8c) in the Jiayuguan gaseous radon with the increased epicenter distance of 343 km.

**Figure 9:**
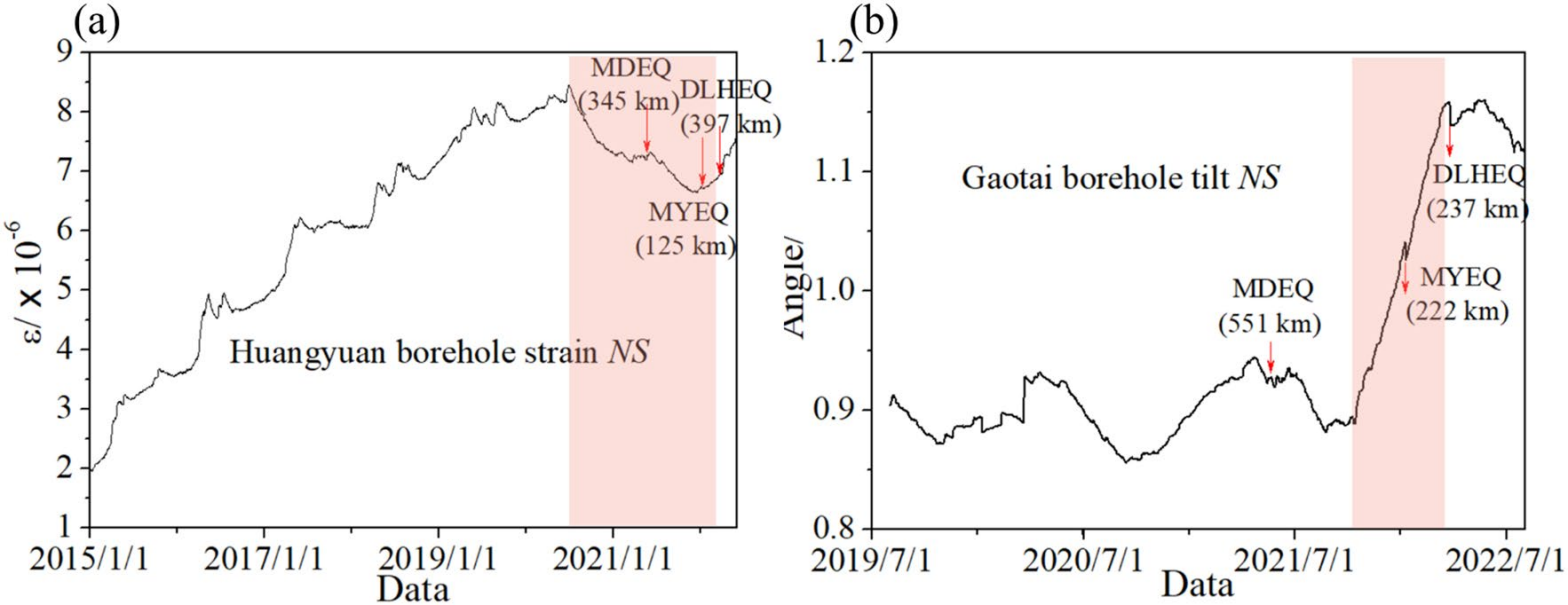
The geodetic time series observed near the hydrological observation stations. (**a**) The *NS* component of the Huangyuan borehole strain was compressed by 1.8 × 10^–6^ right before the MDEQ and MYEQ. The curves in both (**a**) and (**b**) returned to normal after the DLHEQ. (**b**) The *NS* component of the Gaotai borehole tilt was accelerated to tilt northward from October 15, 2021, and reached 0.15 degrees before the MYEQ and DLHEQ.

The hydrological changes before the mainshocks could be evidenced by the geodetic time series observed in the same neighborhoods and during the same period (Fig. 9). The Huangyuan borehole strain, which is located south of the Qilian block, was compressed sharply months before the earthquakes on both Bayan Har and Qilian blocks (e.g., the MDEQ and MYEQ), While the Gaotai Borehole tilt, which is on the north side of the Qilian block (Fig. 2), was accelerated to tilt northward just before the MYEQ.

The tectonics-related hydrological changes have earlier been detected by Sun et al.^24^. They revealed that the anomalous changes of the water temperature in Yushu well correlate well with the large earthquakes on the Tibetan block. When the observation station and the detection earthquake are on the same active tectonic block, significant hydrological changes can be observed before the mainshock due to the crustal stress accumulation in the source media. However, the results presented in this paper further show that when the tectonic block of the observation station is different from that of the detection earthquake, the sensitivity of the hydrological responses attenuated, gradually.

**Figure 10:**
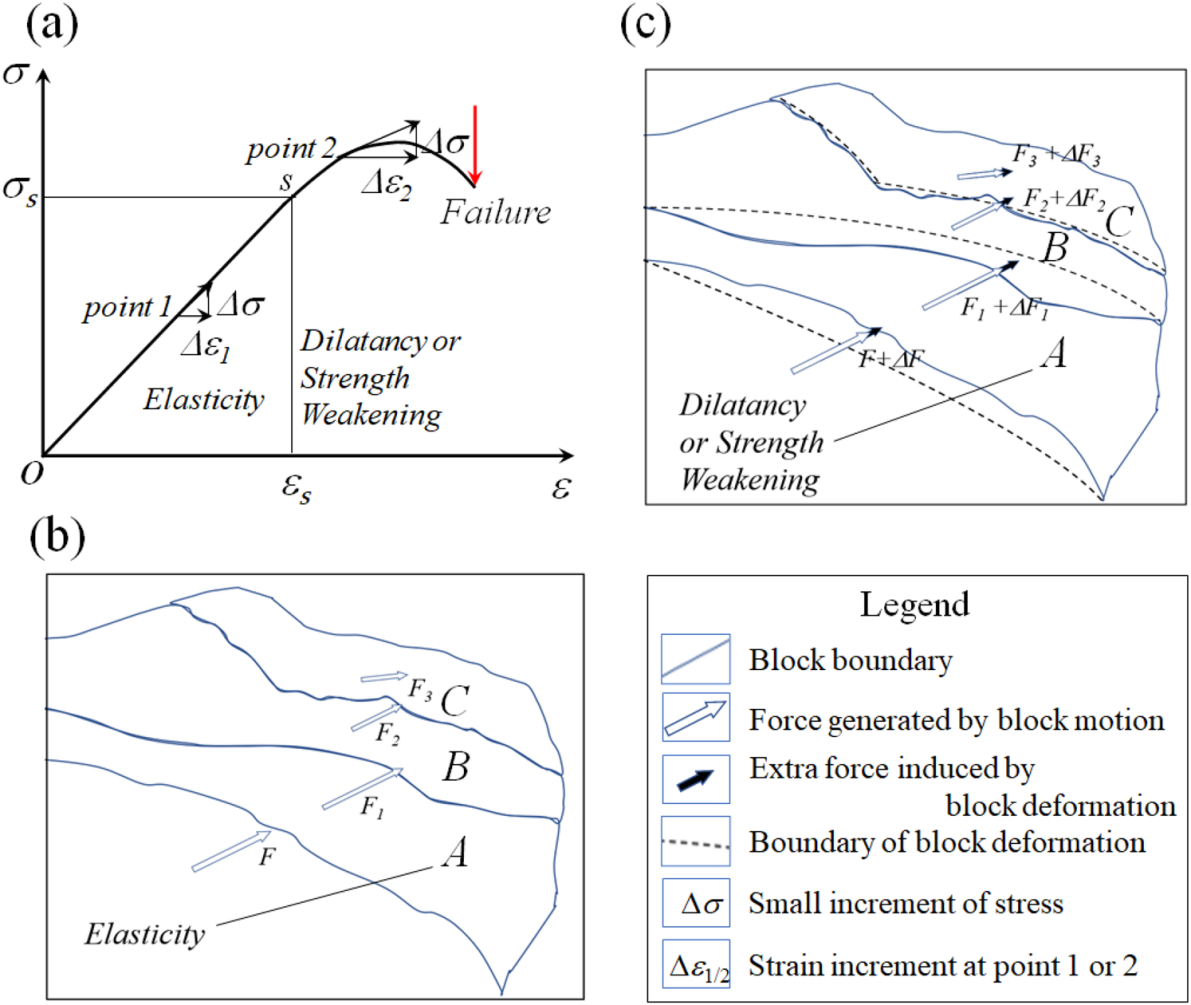
Schematic diagram of the influences of A block on its surrounding blocks during the preparation for an earthquake. (**a**) The constitutive law of a rock, depicting the stress–strain evolution of A block. The strain increment derived from a small increment of stress at the stage of dilatancy or strength weakening is greater than that at the elasticity. (**b**) A Block is in the elastic state. The stresses between the blocks are derived from their movements. (**c**) A Block is in the dilatancy or strength weakening process. The crustal deformation of A block enhances stresses in its surrounding blocks.

Similar to the short-term precursors suggested by Scholz et al.^35^, the temporal and spatial evolutions of the hydrological changes before the MDEQ, MYEQ, and DLHEQ (Figs. 3 and 4) should be derived from the stress–strain relationship of a rock system^36^ (Fig. 10a). When the tectonic stress is low, i.e., the system is in the elastic stage, earthquakes are difficult to be triggered; when the tectonic stress is high, i.e., the system is in the phase of dilatancy, any small disturbance may trigger earthquakes. The observed hydrological responses, such as the decreases in the Gonghe groundwater temperature, increases in the Ledu gaseous radon, and anomalous annual variation in the Pingan groundwater level should therefore be created by the variation of porosity and permeability in source media^22^. In terms of the studies of Kawabata et al.^37^ and Woith et al.^38^, these changes can all be adopted as indicators for testing future large earthquakes.

The modulation of the tectonic block on the hydrological changes is also closely relevant to the evolution of the crustal stress field^39,40^. Because of the complexity of tectonic stress and heterogeneity of source media, the stress accumulation in each of the blocks is inconsistent. Some of them might be critically loaded (where earthquakes tend to occur), while others are not, leading to the difference in the porosity and permeability, which determines the hydrological responses in each block. During the preparation for an earthquake, if A block is critically loaded (Fig. 10a), in addition to the force generated by block motion (Fig. 10b), the extra force derived from the deformation of the block caused by the dilatancy or strength weakening (as the variation of strain increments from point 1 to point 2) augment the stresses in its adjacent blocks (Fig. 10c). Thereby, the pre-seismic changes may also be recorded by the stations on these blocks, such as the changes in the Gonghe groundwater temperature before the MDEQ, MYEQ, and DLHEQ. However, the sensitivity of hydrological response should be decreased due to the friction and energy dissipation between the blocks. This is even more serious for the separated blocks, such as the A and C blocks. Among the stations on the Qilian block, just only the Zuoshu groundwater level manifested some anomalies before the MDEQ (which is located on the Bayan Har block), while for the stations on the Bayan Har block, such as the Yushu groundwater temperature, the precursor can hardly be found before the earthquakes that occurred on the Qilian block (e.g., the MYEQ). Knowing the unique characteristic of the tectonic block-related hydrological changes, we may adopt that for detecting more earthquakes in the future.

## Conclusions

We revealed that the hydrological changes observed in Qinghai province correlate well with the earthquakes in this region in recent years. This observation is valuable for exploring hydrological responses during the seismogenic process. By using the empirical method of short-term precursor monitoring, the potential of future large earthquakes within a certain spatial scope may be assessed. More importantly, we found that the performance of the pre-seismic changes is strongly modulated by the active tectonic blocks. This result augments the understanding of the stress interaction between active tectonic blocks during the establishment of criticality, which is constructive for the countries such as China that are carrying out earthquake prediction tasks. By using this strategy, in practice, we can evaluate the potential of a future earthquake with much more confidence. Meanwhile, it is possible to estimate earthquake location by exploring earthquake-related precursory information through hydrological observation networks.

## Data Availability

The GPS data in Fig. 1 were retrieved from Wang and Shen^27^. The location of the observation stations in Fig. 2, the hydrological data in Figs. 3, 4, 7 and 8, and the geodetic time series in Fig. 9 were from the CENC. The earthquake catalog and focal mechanisms were also from the CENC. The datasets analyzed during the current study are available in the Mendeley data repository (https://data.mendeley.com/datasets/2fhw7c28f4/1).
